# Carotid intraplaque neovascularization increases the risk of white matter hyperintensity progression: a prospective cohort study

**DOI:** 10.3389/fnins.2026.1768896

**Published:** 2026-02-10

**Authors:** Hao Zhang, Jun He, Jimei Xu, Rongfeng Wang, Yan Yan, Yuan Feng, Shugang Cao, Mingwu Xia

**Affiliations:** 1 Department of Neurology, Hefei Hospital Affiliated to Anhui Medical University, The Second People's Hospital of Hefei, Hefei, China; 2 Department of Ultrasound, Hefei Hospital Affiliated to Anhui Medical University, The Second People's Hospital of Hefei, Hefei, China

**Keywords:** AngioPLUS technology, carotid plaque, cerebral small vessel disease, intraplaque neovascularization, white matter hyperintensities progression

## Abstract

**Background and purpose:**

White matter hyperintensities (WMH) are a common imaging manifestation of cerebral small vessel disease (CSVD). While carotid intraplaque neovascularization (IPN) has been implicated in WMH burden, its longitudinal impact on WMH progression, particularly across different brain regions, remains to be fully elucidated.

**Methods:**

We conducted a longitudinal cohort study of patients with baseline WMH, who underwent carotid IPN grading (Grade 0–2) via AngioPLUS ultrasound. Clinical demographics, vascular risk factors, and key laboratory parameters (including homocysteine and LDL-C) were collected. WMH progression was defined as an increase in the total score of the visual Rotterdam Progression Scale (range: −7 to +7) by ≥1 point on follow-up MRI, indicating new or enlarged lesions. Logistic regression analysis was used to determine the relationship between IPN and progression of white matter hyperintensities.

**Results:**

In this longitudinal cohort study, a total of 213 patients (mean age 71.0 ± 8.9 years; 56.8% male) were included in the final analysis. Over a mean follow-up period of 17.8 months (range: 4–52 months), overall progression of white matter hyperintensities (WMH) was observed in 124 patients (58.2%). Specifically, progression of periventricular WMH (PWMH) and deep WMH (DWMH) occurred in 42.3 and 33.8% of patients, respectively. Patients with WMH progression showed a significantly higher prevalence of vascular risk factors compared to those without progression. Logistic regression analysis, after adjustment for age, smoking, alcohol consumption, hypertension, diabetes, history of stroke, body mass index, low-density lipoprotein cholesterol, and follow-up duration, indicated that grade 2 (high-grade) intraplaque neovascularization in the carotid artery was independently associated with the progression of deep white matter hyperintensities (OR 2.06, 95% CI 1.05–4.05).

**Conclusion:**

Carotid intraplaque neovascularization is an independent predictor of deep WMH progression. Assessing plaque vulnerability via IPN grading may help identify patients at high risk for progressive white matter injury and serve as a potential target for therapeutic intervention.

## Introduction

White matter hyperintensities (WMH) are prevalent neuroimaging manifestations characterized by hyperintense signals in the periventricular or subcortical white matter on T2-weighted or fluid-attenuated inversion recovery (FLAIR) sequences. These lesions arise from heterogeneous etiologies and are anatomically classified into periventricular white matter hyperintensities (PWMH) and deep white matter hyperintensities (DWMH) (Wardlaw et al., 2013; Wolters et al., 2017). Accumulating evidence has established advanced age, hypertension, atherosclerosis, dyslipidemia, hyperhomocysteinemia, and hyperuricemia as key risk factors for WMH development (Cox et al., 2019; Liao et al., 2015).

Recent research has increasingly focused on the role of carotid atherosclerosis in cerebral small vessel disease. Specifically, carotid intraplaque neovascularization (IPN)—a hallmark of plaque vulnerability—has been associated with the severity and distribution of WMHs, with a notable predilection for DWMH (Pan et al., 2024; Wang et al., 2020). However, the longitudinal impact of carotid IPN on the *progression* of WMHs remains poorly characterized. Intraplaque neovascularization (IPN) is a critical hallmark of atherosclerotic plaque vulnerability. Its formation originates from the hypoxic and chronically inflammatory microenvironment within the plaque core, which drives the proliferation of vasa vasorum from the adventitia toward the plaque center. However, these neovessels are structurally immature and hyperpermeable, rendering them prone to rupture or leakage. This instability can trigger intraplaque hemorrhage, amplify local inflammatory responses, and release pro-thrombotic materials, such as cholesterol crystals and micro-emboli, into the circulation. Consequently, IPN serves as a pivotal pathological link between local carotid plaque instability and distal cerebrovascular micro-embolic events.

Accurate assessment of IPN is crucial for clinical risk stratification. Ultrasound is the primary non-invasive modality for IPN evaluation. Contrast-enhanced ultrasound (CEUS) allows for the semi-quantification of neovascularization through microbubble enhancement, while superb microvascular imaging (SMI), such as the AngioPLUS technology employed in this study, offers a contrast-agent-free alternative by optimizing the detection of low-velocity blood flow signals, thereby facilitating the visualization of IPN. The validity of this technique has been established in prior studies. Utilizing this advanced ultrasonographic approach, the present study aims to prospectively investigate the specific impact of IPN on the longitudinal progression of white matter hyperintensity subtypes, thereby elucidating its role in the progression of cerebral small vessel disease.

## Patients and methods

### Study population

This longitudinal observational study consecutively enrolled patients admitted to the Department of Neurology at The Second People’s Hospital of Hefei between September 1, 2020, and July 30, 2024. The study protocol was approved by the Ethics Review Committee of The Second People’s Hospital of Hefei and was conducted in accordance with the Declaration of Helsinki. Written informed consent was obtained from all participants or their legal guardians.

Inclusion criteria were as follows: (1) underwent assessment of carotid IPN using AngioPLUS ultrasound technology; and (2) completed baseline and follow-up cranial magnetic resonance imaging (MRI) with assessable WMH burden.

Exclusion criteria included: (1) severe carotid artery stenosis (defined as ≥70% stenosis); (2) presence of extensive calcified plaques precluding IPN evaluation; (3) history of intracerebral hemorrhage or large-territory ischemic infarction; (4) acute ischemic stroke occurring at baseline or during the follow-up period were excluded; (5) history of or concurrent malignancy; (6) severe dementia or behavioral disorders preventing cooperation; (7) atrial fibrillation or other cardioembolic sources; and (8) incomplete clinical/imaging data or suboptimal image quality.

### Clinical data collection

**Figure 1:**
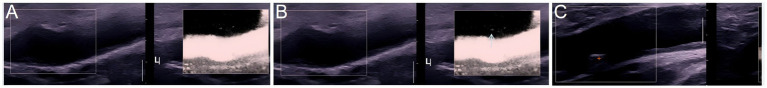
Carotid IPN grading. **(A)** Grade 0 IPN; **(B)** Grade 1 IPN; **(C)** Grade 2 IPN. In each panel, the left portion displays the two-dimensional B-mode ultrasound image, and the right portion shows the corresponding AngioPLUS imaging visualizing intraplaque microvascular flow signals.

**Figure 2:**
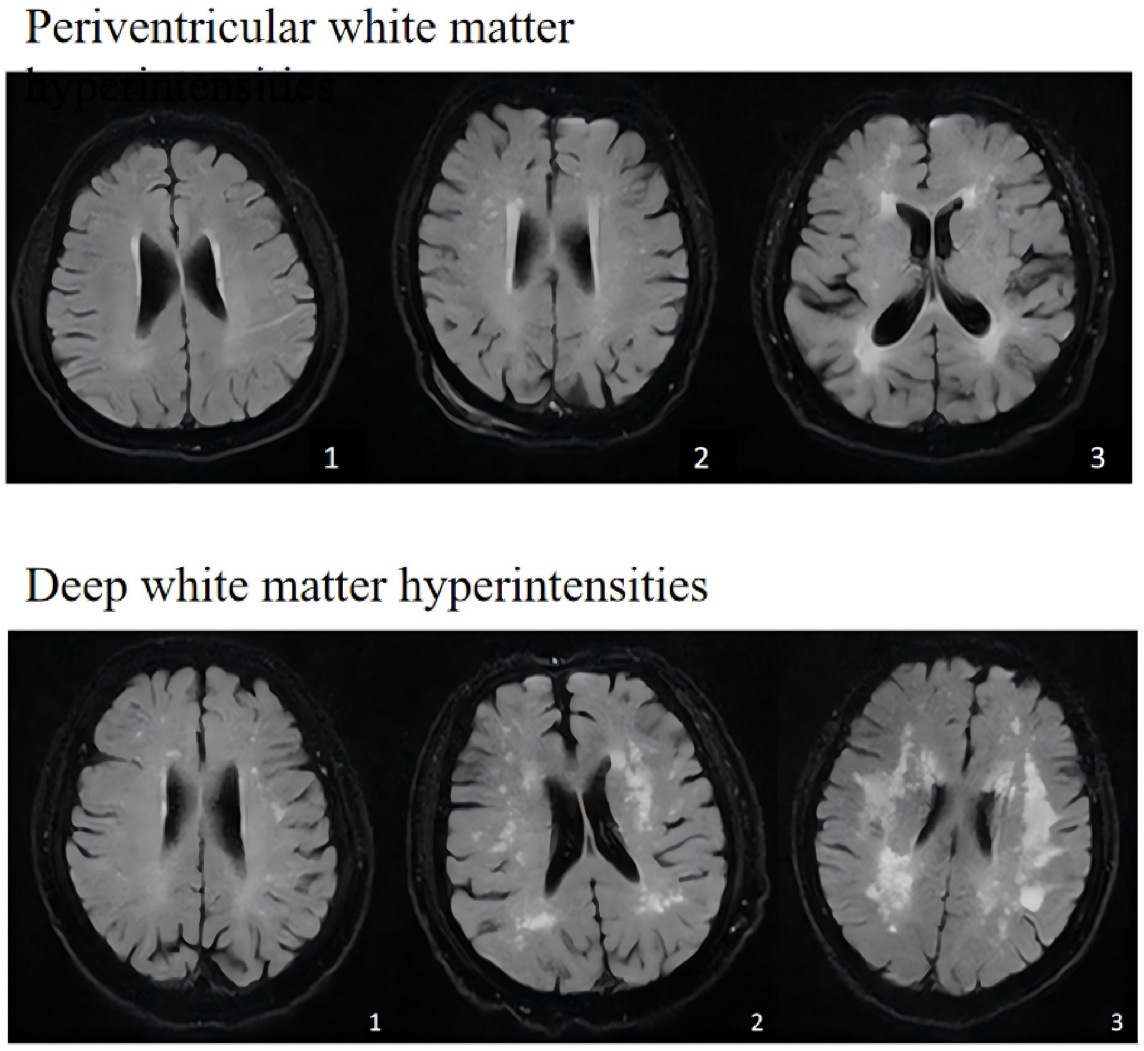
Imaging manifestations of WMH across different Fazekas scores.

Comprehensive demographic data (age, sex) and vascular risk profiles were collected upon admission. Risk factors included current smoking, alcohol consumption, hypertension, diabetes mellitus, and history of transient ischemic attack (TIA) or ischemic stroke. Body mass index (BMI) was calculated using standard protocols (see Figures 1, 2).

### Laboratory investigations

Fasting venous blood samples were obtained from all participants the morning after admission. A complete blood count was performed using a Sysmex XN-10 automated hematology analyzer. Biochemical parameters, including triglycerides (TG), total cholesterol (TC), high-density lipoprotein cholesterol (HDL-C), low-density lipoprotein cholesterol (LDL-C), fasting blood glucose (FBG), and homocysteine (Hcy), were quantified using an automated biochemical analyzer (HITACHI 7600–020, Japan).

### Carotid plaque diagnosis and IPN evaluation

Carotid arteries were initially evaluated using conventional B-mode ultrasonography. Upon detection of a plaque, contrast-enhanced ultrasound or microflow imaging (AngioPLUS) was performed using a SuperSonic Imagine AixPlorer system (Aix-en-Provence, France) equipped with an SL10-2 linear array transducer. This modality allows for the visualization of microvascular flow signals indicative of IPN (Zhang et al., 2017). In patients with multiple plaques, the thickest plaque was selected as the target for analysis.

IPN severity was graded based on the visual characteristics of microflow signals within a 2-min video clip, as previously described (Chen et al., 2022; Zamani et al., 2019):

- Grade 0: No detectable flow signals within the plaque.
- Grade 1: Limited flow signals (spots or short lines, < 4 signals) confined to the plaque adventitial side or shoulder.
- Grade 2: Extensive flow signals (dendritic or diffuse linear patterns) extending into the plaque core.

For statistical analysis, IPN status was dichotomized into Low IPN (Grades 0–1) and High IPN (Grade 2). Two experienced sonographers, blinded to the patients’ clinical and MRI data, independently performed the evaluations. Discrepancies were resolved by consensus with a third senior sonologist. The inter-rater agreement, expressed by Cohen’s kappa (ĸ), was substantial for total progression at follow-up (0.72).

### MRI acquisition and scoring

MR images were obtained with a1.5 Tesla (T). For this study standard axial T2-weighted fast spin echo and fluid attenuated inversion recovery sequences were used. Scanning parameters were unchanged between baseline and follow-up. All patients underwent both the carotid IPN ultrasound assessment and the baseline cranial MRI within the same hospitalization or outpatient evaluation period, with an interval of no more than 3 days between the two examinations.

#### Baseline assessment

The burden of WMH was assessed using the Fazekas scale (range 0–3 for each region) (Fazekas et al., 1987). PWMH and DWMH were scored separately:

- PWMH: 0 = absent; 1 = caps or pencil-thin lining; 2 = smooth halo; 3 = irregular hyperintensities extending into the deep white matter.
- DWMH: 0 = absent; 1 = punctate foci; 2 = beginning confluence; 3 = large confluent areas.

#### Progression assessment

WMH progression on follow-up MRI was evaluated using the Rotterdam Progression Scale proposed by Prins et al. (2004). This scale measures changes in three periventricular regions (frontal caps, occipital caps, and bands) and four subcortical regions (frontal, parietal, temporal, and occipital). An increase in WMH burden in any region (defined as new lesions or enlargement of existing lesions) was assigned a score of +1. Progression scores ranged from −3 to +3 for periventricular regions and −4 to +4 for deep regions. In this study, WMH progression was defined as a total progression score ≥ 1. Two board-certified vascular neurologists, blinded to clinical and ultrasound data, independently evaluated the MRI sequences. The inter-rater agreement, expressed by Cohen’s kappa (ĸ), was substantial for total progression at follow-up (0.79).

### Statistical analysis

Data analysis was performed using R software (version 4.5.1; R Foundation for Statistical Computing, Vienna, Austria). Continuous variables were assessed for normality using the Shapiro–Wilk test and are presented as mean ± standard deviation (SD) or median [interquartile range (IQR)], as appropriate. Between-group comparisons were conducted using the Student’s *t*-test or Mann–Whitney U test. Categorical variables are expressed as frequencies (percentages) and were compared using the Chi-square test or Fisher’s exact test.

The relationship between carotid IPN and the progression of WMH was assessed using binary logistic regression analysis. We performed three separate analyses: PWMH progression, DWMH progression and progression of WMH in any (periventricular and/or deep) region, respectively. All models were adjusted for cardiovascular risk factors—including age, smoking, alcohol consumption, hypertension, diabetes mellitus, history of stroke, HDL, LDL, and BMI—as well as follow-up duration. Odds ratios (OR) are given with 95% confidence interval (CI). Statistical significance was considered at *p* < 0.05.

## Results

### Study population and baseline characteristics

Of the 245 patients initially enrolled with carotid plaque and WMH, 32 were excluded due to poor ultrasound image quality (*n* = 7), loss to follow-up (*n* = 10), cardioembolic sources (*n* = 3), malignancies (*n* = 3), moyamoya disease (*n* = 3), or incomplete clinical/imaging data (*n* = 6). Consequently, the final analysis included 213 patients (121 men, 56.8%; mean age 71.0 ± 8.9 years). The mean follow-up duration was 17.8 months (range: 4–52 months).

During the follow-up period, 124 patients (58.2%) exhibited progression of WMH, either in periventricular regions, deep regions, or both. Specifically, progression of PWMH occurred in 90 patients (42.3%), while DWMH progressed in 72 patients (33.8%). As presented in Table 1, compared with the non-progression group, patients with overall WMH progression had a significantly higher prevalence of smoking, alcohol consumption, and history of ischemic stroke, a longer follow-up duration, and elevated LDL levels (*p* < 0.05). Subgroup analysis revealed that smoking, history of ischemic stroke, and longer follow-up time were associated with PWMH progression (*p* < 0.05). In contrast, DWMH progression was significantly associated with a history of ischemic stroke, higher BMI, and higher LDL levels (*p* < 0.05).

**Table 1:** Baseline characteristics of patients (*N* = 213).

Characteristics	All patients(*N* = 213)	No WMH progression (*n* = 89)	Any WMH Progression (*n* = 124)	*p*-value	No PWMH progression (*n* = 123)	PWMH progression (*n* = 90)	*p*-value	No DWMH progression (*n* = 141)	DWMH progression (*n* = 72)	*p*-value
Age, year, mean ± SD	71.02 ± 8.857	71.39 ± 9.131	70.90 ± 8.506	0.831	71.46 ± 9.00	70.62 ± 8.433	0.581	70.86 ± 8.761	71.60 ± 8.783	0.492
Sex, man, *n* (%)	121 (56.8)	51 (57.30)	70 (56.45)	0.901	70 (56.91)	51 (56.67)	0.972	80 (56.74)	41 (56.94)	0.977
Smoking, *n* (%)	40 (18.78)	10 (11.24)	30 (24.19)	0.017	17 (13.82)	23 (25.56)	0.03	22 (15.60)	18 (25)	0.097
Drinking, *n* (%)	33 (15.49)	8 (8.99)	25 (20.16)	0.026	16 (13.01)	17 (18.89)	0.241	18(12.77)	15 (20.83)	0.124
Hypertension, *n* (%)	165 (77.46)	74 (83.15)	91 (73.39)	0.093	101 (82.11)	64 (71.11)	0.058	111 (78.72)	54 (75)	0.538
Diabetes, *n* (%)	64 (30.05)	26 (29.21)	38 (30.65)	0.822	35 (28.46)	29(32.22)	0.554	42 (29.79)	22 (30.56)	0.908
History of stroke, *n* (%)	56 (26.29)	15 (16.85)	41 (33.06)	0.008	22 (17.89)	34 (37.78)	0.001	31 (21.99)	25 (34.72)	0.046
BMI, mean ± SD	24.388 ± 2.897	24.11 ± 3.134	24.59(2.708)	0.229	24.33 ± 2.97	24.463 ± 2.806	0.748	24.10 ± 3.05	24.956 ± 2.499	0.029
Hcy, median (IQR)	12.5 (10.6, 16.1)	11.90 (10.30, 14.85)	12.8 (10.7,16.4)	0.154	12.1 (10.2, 15.4)	13.15 (10.70, 16.425)	0.165	12.30 (10.60, 15.95)	12.8 (10.625, 16.4)	0.442
TC, mean ± SD	4.04 (3.39, 4.795)	3.97 (3.41, 4.92)	4.12 (3.32, 4.668)	0.953	4.01 (3.40, 4.90)	4.125 (3.288, 4.623)	0.968	4.02 (3.355, 4.820)	4.135 (3.413, 4.748)	0.490
TG, median (IQR)	1.31 (0.91, 1.845)	1.44 (1.0, 1.89)	1.25 (0.89, 1.745)	0.148	1.27 (0.89, 1.89)	1.32 (0.950, 1.7625)	0.900	1.31 (0.925, 1.835)	1.3 (0.89, 1.88)	0.935
HDL, mean ± SD	1.22 (1.055, 1.435)	1.26 (1.08, 1.525)	1.20 (1.04, 1.408)	0.081	1.26 (1.09, 1.47)	1.175 (1.03, 1.4225)	0.056	1.23 (1.065, 1.46)	1.205 (1.04, 1.403)	0.250
LDL, median (IQR)	2.23 (1.61, 2.865)	2.16 (1.425, 2.7)	2.37 (1.78, 2.93)	0.031	2.19 (1.56, 2.89)	2.265 (1.678, 2.853)	0.335	2.09 (1.49, 2.68)	2.54 (1.925, 3.255)	<0.001
FBG, median (IQR)	5.21 (4.725, 6.205)	5.14 (4.67, 5.925)	5.26 (4.79, 6.41)	0.331	5.18 (4.63, 5.87)	5.275 (4.858, 6.625)	0.108	5.19 (4.67, 6.00)	5.265 (4.825, 6.530)	0.232
IPN, *n* (%)				0.585			0.209			0.225
Grades 0 and 1, *n* (%)	106 (49.77)	44 (49.44)	62 (50)		59 (47.97)	51 (56.67)		77 (54.61)	33 (45.83)	
Grades 2, *n* (%)	107 (50.23)	45 (50.56)	62 (50)		64 (52.03)	39 (43.33)		64 (45.39)	39 (54.17)	
Follow-up time	13 (10, 22)	12 (9, 17.25)	12(10, 21)	0.008	12 (10, 18)	15.5 (11, 33)	0.002	12 (10, 21)	13 (11.5, 30)	0.123

BMI, body mass index; Hcy, homocysteine; TC, total cholesterol; TG, triglyceride; HDL, high-density lipoprotein; LDL, low-density lipoprotein; FBG, fasting blood glucose; IPN, intraplaque neovascularization; WMH, White matter hyperintensities; PWMH, periventricular white matter hyperintensities; DWMH, deep white matter hyperintensities.

### Carotid IPN and WMH progression

To evaluate the presence of multicollinearity among the covariates included in the model, the Variance Inflation Factor (VIF) was calculated for each variable (Table 2). The VIF values ranged from 1.10 to 2.00, which are considerably below the commonly accepted thresholds of 5 or 10. These findings confirm the absence of significant multicollinearity, ensuring the reliability of the regression estimates.

Binary logistic regression analysis demonstrated that, after adjusting for age, smoking, alcohol consumption, hypertension, diabetes, history of stroke, body mass index, low-density lipoprotein cholesterol, and follow-up duration, grade 2 intraplaque neovascularization in the carotid artery was associated with the progression of deep white matter hyperintensities (OR 2.06, 95% CI 1.050–4.050, Table 3). Given the considerable variation in follow-up time among the study population, sensitivity analyses were performed. The association remained significant after excluding participants with a follow-up duration of less than 6 months (OR 2.11, 95% CI 1.040–4.310; Table 4) and further after excluding those with less than 12 months of follow-up (OR 2.89, 95% CI 1.21–6.91; Table 4). However, in the regression analyses, grade 2 intraplaque neovascularization showed no significant association with the progression of either overall white matter hyperintensities or periventricular white matter hyperintensities (see Table 5).

**Table 2:** Variance inflation factor (VIF) results.

Variable	VIF value
Age	1.125
Smoking	1.642
Drinking	1.589
Hypertension	1.171
Diabetes	1.102
History of stroke	1.152
BMI	1.143
HDL	1.162
LDL	1.186
Follow-up time	1.261

**Table 3:** Binary logistic regression analysis with progression of white matter hyperintensities as dependent variable (*N* = 213).

Characteristics	Any WMH progression^a^	*p*	Periventricular WMH progression^b^	*p*	Deep WMH progression^c^	*p*
Grades 2, *n* (%)	1.22 (0.65–2.28)	0.532	0.97 (0.51–1.82)	0.927	2.06 (1.050–4.050)	0.036

a Multivariable adjustment for age, smoking, alcohol consumption, hypertension, diabetes, stroke history, HDL, LDL, and follow-up time.

b Multivariable adjustment for age, smoking, alcohol consumption, hypertension, diabetes, stroke history, HDL, and follow-up time.

c Multivariable adjustment for age, smoking, alcohol consumption, hypertension, diabetes, stroke history, BMI, LDL, and follow-up time.

WMH, White matter hyperintensities; PWMH, periventricular white matter hyperintensities; DWMH, deep white matter hyperintensities.

**Table 4:** Binary logistic regression analysis with progression of white matter hyperintensities as dependent variable (*N* = 200, the follow-up time was 6 months or more).

Characteristics	Any WMH progression^a^	*p*	Periventricular WMH progression^b^	*p*	Deep WMH progression^c^	*p*
Grades 2, n (%)	1.11 (0.58–2.14)	0.756	0.84 (0.44–1.59)	0.589	2.11 (1.040–4.31)	0.040

a Multivariable adjustment for age, smoking, alcohol consumption, hypertension, diabetes, stroke history, HDL, LDL, and follow-up time.

b Multivariable adjustment for age, smoking, alcohol consumption, hypertension, diabetes, stroke history, HDL, and follow-up time.

c Multivariable adjustment for age, smoking, alcohol consumption, hypertension, diabetes, stroke history, BMI, LDL, and follow-up time.

WMH, White matter hyperintensities; PWMH, periventricular white matter hyperintensities; DWMH, deep white matter hyperintensities.

**Table 5:** Binary logistic regression analysis with progression of white matter hyperintensities as dependent variable (*N* = 138, the follow-up time was 12 months or more).

Characteristics	Any WMH progression^a^	*p*	Periventricular WMH progression^b^	*p*	Deep WMH progression^c^	*p*
Grades 2, n (%)	1.65 (0.71–3.81)	0.234	0.89 (0.40–2.00)	0.777	2.89 (1.21–6.91)	0.017

a Multivariable adjustment for age, smoking, alcohol consumption, hypertension, diabetes, stroke history, HDL, LDL, and follow-up time.

b Multivariable adjustment for age, smoking, alcohol consumption, hypertension, diabetes, stroke history, HDL, and follow-up time.

c Multivariable adjustment for age, smoking, alcohol consumption, hypertension, diabetes, stroke history, BMI, LDL, and follow-up time.

WMH, White matter hyperintensities; PWMH, periventricular white matter hyperintensities; DWMH, deep white matter hyperintensities.

## Discussion

In this longitudinal cohort study, WMH progression was observed in 58.2% of patients over a follow-up period ranging from 4 to 52 months. The primary finding was that high-grade intraplaque neovascularization (IPN grade 2) in the carotid artery served as an independent predictor of DWMH progression. This association remained robust in sensitivity analyses that sequentially excluded participants with follow-up durations of less than 6 months and less than 12 months, confirming the independent predictive value of IPN grade 2 for deep white matter hyperintensity progression.

Our findings align with and extend previous research linking carotid atherosclerosis to cerebral small vessel disease. While plaque-induced stenosis is known to alter hemodynamics and increase arterial pulsatility transmitted to cerebral small vessels (Kwak et al., 2014; Liao et al., 2015), recent evidence suggests that plaque vulnerability plays a more specific role. Wang et al., (2020) previously demonstrated an association between carotid IPN and WMH severity, patients with IPN grade 3 had a 25.84-fold higher likelihood of a high WMH burden score compared to those with IPN grade 0, and Li et al. (2024), using high-resolution MRI, identified intraplaque hemorrhage (IPH)—a consequence of IPN—as a risk factor for WMH in acute stroke patients. Our study corroborates these cross-sectional associations and provides longitudinal evidence that IPN actively accelerates DWMH progression.

Several non-exclusive, pathophysiological mechanisms may potentially explain the observed link between carotid IPN and DWMH progression. First, microembolism originating from unstable plaques is one plausible pathway. Neovessels within atherosclerotic plaques are structurally immature and fragile, which could make them prone to rupture and lead to intraplaque hemorrhage. The subsequent release of microemboli might occlude distal penetrating arterioles (Altaf et al., 2006; Berman et al., 2015). The deep white matter, supplied by long penetrating arteries with limited collateral circulation, is thought to be particularly susceptible to such repeated microembolic insults, the cumulative effect of which may contribute to the progression of DWMH over time. Second, IPN may be associated with enhanced systemic and local inflammation. Neovessels can serve as conduits for inflammatory cell infiltration into the plaque, potentially transforming it into a source of proinflammatory cytokines (Zhang et al., 2019). These circulating mediators might compromise the blood–brain barrier integrity, allowing leakage of plasma components into the white matter. This process could trigger neuroinflammation and oxidative stress, which are implicated in oligodendrocyte injury and myelin damage (Alateeq et al., 2024; Gertje et al., 2023). Additionally, exosomal signaling has been proposed as another novel mechanistic avenue; unstable plaques may release exosomes containing pathogenic microRNAs that could modulate glial cell function and potentially impair white matter integrity (Zhang et al., 2025). Third, chronic cerebral hypoperfusion might play a contributory role. The deep white matter resides in arterial watershed zones and is inherently vulnerable to reductions in perfusion. IPN, as a marker of advanced atherosclerosis and plaque instability, may correlate with a compromised cerebral perfusion reserve (Damestani et al., 2024; Muer et al., 2024; Sowa et al., 2015). In the context of hemodynamic compromise, the distal deep white matter might experience a chronic mismatch between metabolic supply and demand, possibly leading to gradual axonal degeneration and lesion expansion. It is important to emphasize that these mechanisms remain speculative within the context of our present study and warrant further investigation through targeted, multimodal prospective research to establish causal pathways.

### Limitations

Several limitations should be acknowledged. First, this was a single-center study with a relatively small sample size, which limits generalizability. Second, we focused on WMH and did not assess other imaging markers of cerebral small vessel disease, such as cerebral microbleeds or enlarged perivascular spaces. Future multi-center studies with comprehensive multimodal imaging are warranted to validate these findings. Finally, in this study, both baseline and follow-up cranial MRI examinations were performed based on clinical indications rather than at pre-specified, uniform intervals according to a prospective research protocol. Although this design aligns more closely with real-world clinical practice, it may have introduced heterogeneity in follow-up timing and potentially affected the precise estimation of the rate of white matter hyperintensity progression. Additionally, while the visual rating scales employed in this study ensure clinical comparability and practicality, their sensitivity to early-stage or subtle white matter changes is limited. Currently, machine learning-based automated whole-brain WMH segmentation and volumetric quantification techniques offer higher sensitivity and objectivity, enabling precise measurement of WMH progression rates and spatial distribution patterns. The application of such automated methods may allow earlier detection of WMH evolution and reveal more nuanced associations between carotid IPN and white matter injury. Future multicenter prospective cohort studies incorporating automated quantification and multimodal neuroimaging are needed to further validate the findings of this study and to explore the dose–response relationship as well as the integrated pathological mechanisms linking IPN to WMH progression.

Our findings establish carotid intraplaque neovascularization (IPN) as an independent risk factor for deep white matter hyperintensity progression, carrying direct clinical relevance. Detection of high-grade IPN by ultrasound aids in risk stratification by identifying patients prone to rapid white matter injury, thereby informing personalized surveillance. This supports a bidirectional screening rationale: assessing carotid plaque vulnerability in individuals with progressive deep white matter lesions, and conversely, considering brain MRI screening in atherosclerosis patients with high-risk IPN. Ultimately, targeting IPN emerges as a promising therapeutic strategy for stabilizing vulnerable plaques and mitigating the progression of cerebral small vessel disease.

## Conclusion

In conclusion, this study demonstrates that carotid intraplaque neovascularization, specifically High-Grade IPN, is a significant and independent risk factor for the progression of DWMH. These findings suggest that evaluating plaque vulnerability via carotid ultrasound may serve as a valuable, non-invasive tool for identifying patients at high risk of progressive cerebral small vessel disease, enabling earlier and more targeted preventive interventions.

## Data Availability

The raw data supporting the conclusions of this article will be made available by the authors, without undue reservation.
